# Interpreting the Pharmacological Mechanisms of Huachansu Capsules on Hepatocellular Carcinoma Through Combining Network Pharmacology and Experimental Evaluation

**DOI:** 10.3389/fphar.2020.00414

**Published:** 2020-04-03

**Authors:** Jihan Huang, Feiyu Chen, Zhangfeng Zhong, Hor Yue Tan, Ning Wang, Yuting Liu, Xinyuan Fang, Tao Yang, Yibin Feng

**Affiliations:** ^1^ Center for Drug Clinical Research, Shuguang Hospital, Shanghai University of Traditional Chinese Medicine, Shanghai, China; ^2^ School of Chinese Medicine, Li Ka Shing Faculty of Medicine, The University of Hong Kong, Hong Kong, Hong Kong; ^3^ Department of Cardiology, Cardiovascular Research Institute, Shuguang Hospital affiliated to Shanghai University of Traditional Chinese Medicine, Shanghai, China; ^4^ Marine College, Shandong University (Weihai), Weihai, China

**Keywords:** Huachansu capsules, network pharmacology, hepatocellular carcinoma, molecular targets, KEGG pathway

## Abstract

Hepatocellular carcinoma (HCC) is one of the most fatal cancers across the world. Chinese medicine has been used as adjunctive or complementary therapy for the management of HCC. Huachansu belongs to a class of toxic steroids isolated from toad venom that has important anti-cancer property. This study was aimed to identify the bioactive constituents and molecular targets of Huachansu capsules (HCSCs) for treating HCC using network pharmacology analysis and experimental assays. The major bioactive components of HCSCs were determined using ultra-performance liquid chromatography-tandem mass spectrometry (UPLC-MS/MS). A series of network pharmacology methods including target prediction, pathway identification, and network establishment were applied to identify the modes of action of HCSCs against HCC. Furthermore, a series of experiments, including MTT, clonogenic assay, 3-D transwell, wound healing assay, as well as flow cytometry, were conducted to verify the inhibitory ability of HCSCs on HCC *in vitro*. The results showed that 11 chemical components were identified from HCSCs. The network pharmacological analysis showed that there were 82 related anti-HCC targets and 14 potential pathways for these 11 components. Moreover, experimental assays confirmed the inhibitory effects of HCSCs against HCC *in vitro*. Taken together, our study revealed the synergistic effects of HCSCs on a systematic level, and suggested that HCSCs exhibited anti-HCC effects in a multi-component, multi-target, and multi-pathway manner.

## Introduction

Hepatocellular carcinoma (HCC) is an aggressive malignancy and the third leading cause of cancer-related death (Colagrande et al., 2016). Up to now, surgical resection is the principal therapy for HCC patients who were diagnosed at the early stage (Daher et al., 2018), but it is not satisfying for those were diagnosed in the middle or later stages. There is first-line systemic treatment such as sorafenib in the clinical practice, which is the first molecular-targeted agent for HCC treatment, and has been approved in Japan for the treatment of unresectable HCC (Daoudaki and Fouzas, 2014; Omata et al., 2017). Although sorafenib has been shown to provide survival benefits for patients, there are many side effects such as fatigue, diarrhea, hypertension, baldness, and hand-foot skin reaction (Li et al., 2015). With the empirical applications and refinements of Chinese medicine for over two thousand years, it becomes a prominent part of the medical system in China. Using alone or in combination with conventional chemo- or radio- therapy, Chinese medicine has been demonstrated to improve immunity, enhance quality of life and progression-free survival in patients with HCC (Huang et al., 2013). Besides, Chinese herbal formulas are widely used in clinical practice due to its multi-component and multi-target for the treatment of cancers.

Huachansu, a class of toxic steroids isolated from toad venom, has a range of properties including detoxification, detumescence, and pain relief (Zhan et al., 2020). It has been widely used in various diseases and ailments such as chronic hepatitis B and cancer (Cui et al., 2010). Although Huachansu has remarkable inhibitory effects against many types of cancer, it is mainly used for the treatment of advanced tumors (Meng et al., 2009). It has been demonstrated that Huachansu has good therapeutic effects against various advanced malignant tumors including HCC, lung, and pancreatic cancer (Meng et al., 2009). Huachansu capsules (HCSCs) have been approved by the China Food and Drug Administration (No. Z20050846) in 2005 and are produced by Shanxi Eastantai Pharmaceutical Corporation Limited. A study reported that when used in combination with conventional chemotherapy, HCSCs could synergistically enhance the efficacy of chemotherapy and reduce its toxicity (Qin et al., 2008).

A range of pharmacological studies of HCSCs revealed that toad venom lactones are the main active ingredients of HCSCs, which contain cinobufagin (Qi et al., 2010), resibufogenin (Xie et al., 2012), and bufalin (Meng et al., 2016). These studies also demonstrated that these three compounds are the major cardiac glycosides that exert the anti-tumor activities of HCSCs. However, the underlying mechanisms of these anti-tumor effects remain unclear because of the complex compositions. Indeed, the complex compositions of Chinese herbal formulas make it difficult to characterize their active substances, the activities of the active substances, and the compatibilities with multiple ingredients, which have the characteristics of multi-target and multi-pathway. The approaches of systematic pharmacology and network pharmacology provide a new perspective for studying Chinese herbal formulas (Zineh, 2019). Our previous studies have successfully predicted the bioactive substances and molecular targets of several Chinese herbal formulas, which included Yinchenhao decoction (Huang et al., 2017), Danlu capsule (Huang et al., 2017a), Xuangui dropping pill (Huang et al., 2017b), and Fructus Schisandrae (Hong et al., 2017).

**Figure 1:**
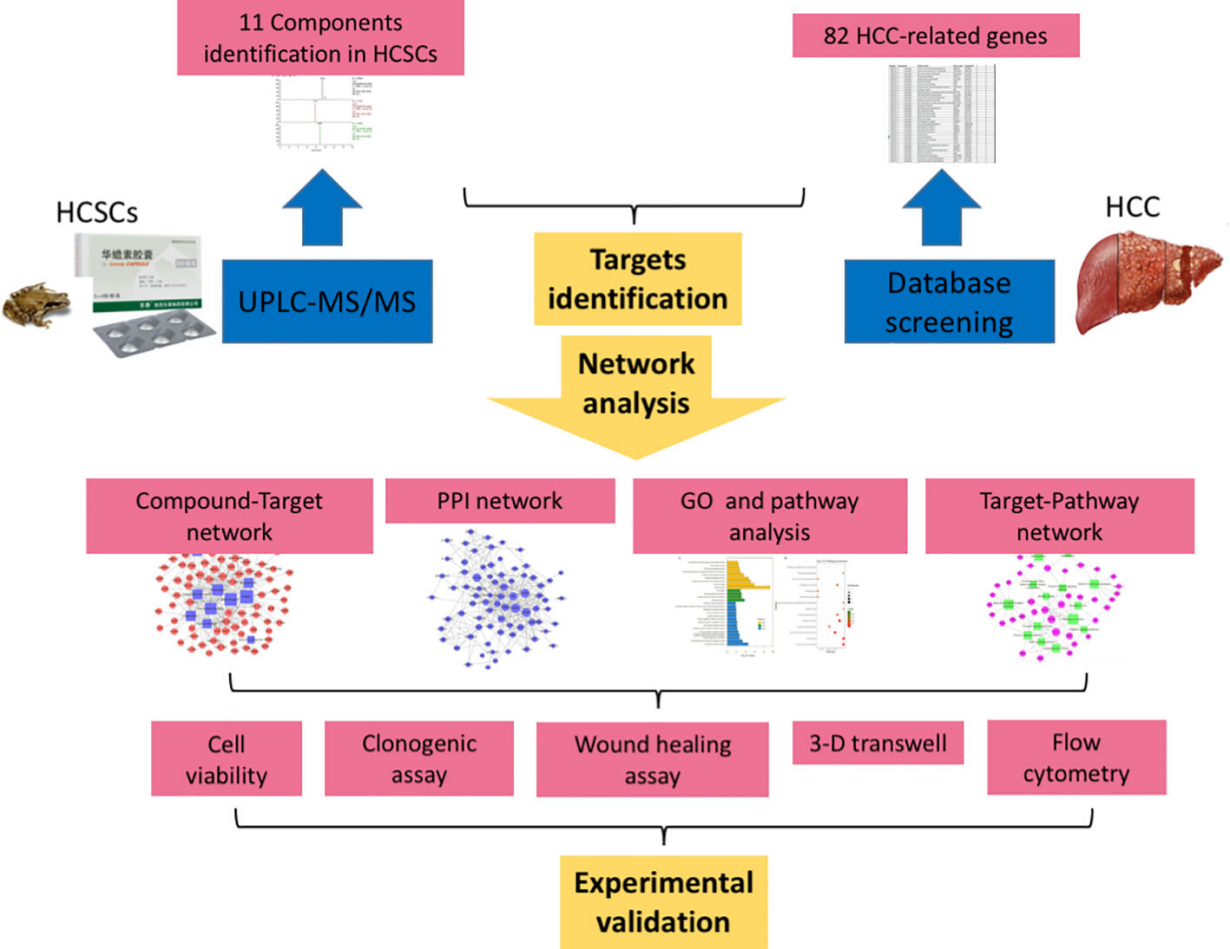
Flowchart of the network pharmacological and experimental studies of Huachansu capsules in hepatocellular carcinoma.

In the current study, we identified the major bioactive components of HCSCs. Moreover, a series of network pharmacological analyses, including target prediction and enrichment, pathway analysis, and network construction, were also conducted to identify the HCC-related targets and potential mechanisms of HCSCs. On the other hand, a series of experimental assays were performed in HCC cell lines, PLC/PRF/5 and MHCC97L, to confirm the inhibitory effects of HCSCs including cell proliferation, colony formation, cell invasion and migration, cell cycle, and cell apoptosis in HCC. The workflow of our network pharmacological and experimental studies of HCSCs in HCC is shown in **Figure 1**. Our results did not only demonstrate the synergistic anti-cancer activities of HCSCs components and their potential targets, but also offer an in-depth knowledge of the molecular mechanisms of HCSCs.

## Methods

### Network Pharmacology-Based Analysis

#### Ultra-Performance Liquid Chromatography-Tandem Mass Spectrometry (UPLC-MS/MS)

The pulverized HCSCs (ca. 0.25 g) was accurately weighed and added to methanol-water (30: 70, v/v; 10 ml). The mixture was processed with ultrasonication for 25 min and centrifugation at 3000 g for 10 min, and then was filtered using a filter (0.25 μm; Millipore, USA). An aliquot (3 μl) of the filtered supernatant was injected for UPLC-Q/ExactiveHybrid Quadrupole-Orbitrap system analysis with the Compound Discoverer 2.1 software package (Thermo Finnigan, San Jose, CA, USA). The detail conditions of the assay are as follows: mobile phase A was 0.1% aqueous solution of formic acid and mobile phase B was acetonitrile. Chromatographic separation was performed on an Acquity UPLC^®^ HSS T3 column (2.1 mm × 100 mm, 1.8 μm); the column temperature was 45°C, flow rate was 0.3 ml/min, and the sample volume was 3 μl. Then the data was analyzed under the model of cation and anion by the means of UHPLC-Q/Exactive, electrospray ionization (ESI) source; scanning model: full MS (resolution 70,000) and ddMS2 (resolution 17,500, NCE35, stepped NCE50%; voltage of the ESI source, +3.2 kV/−2.8 kV). The temperature and voltage of the capillary tube were 320°C and 75.0 V, respectively. The flow rate of the sheath and auxiliary gas were 32.0 and 10.0 arbitrary unit, respectively (scanned area m/z: 80–1,050).

#### Identification of Associated Molecular Targets of HCSCs

The potential molecular targets of HCSCs were predicted using SwissTargetPrediction (Gfeller et al., 2014), similarity ensemble approach (SEA) (Keiser et al., 2007), the Traditional Chinese Medicines for Systems Pharmacology Database and Analysis Platform (TCMSP) (Ru et al., 2014), and the Search Tool for Interacting Chemicals (STITCH) (Szklarczyk et al., 2016).

The HCC-associated human genes were comprehensively retrieved from five databases, which were OncoDB.HCC (Su et al., 2007), Liverome (Lee et al., 2011), Therapeutic Target Database (*TTD*), Kyoto Encyclopedia of Genes and Genomes (KEGG), the Comparative Toxicogenomics Database (CTD), and GeneCards.

#### Protein-Protein Interaction (PPI) Network

The STRING online database was applied to obtain the PPI data of the molecular targets of HCSCs (Szklarczyk et al., 2019), where the parameter organism was set to Homo sapiens, other basic settings were the default value. Cytoscape software was employed to establish the PPI relationship network and perform topological analysis.

#### Gene Ontology (GO) and KEGG Pathway Enrichment Analyses

The GO analysis and KEGG pathway enrichment were employed by using the Database for Annotation, Visualization and Integrated Discovery (DAVID). The biological process (BP), cellular component (CC), and molecular function (MF) in GO were selected to annotate the gene function. Terms with expression analysis systematic explorer scores of ≤0.05 were collected for functional annotation clustering. The pathway enrichment analysis was performed using the KEGG database to verify the functional categories of statistically significant genes (p < 0.05). Terms with thresholds of count of ≥2 and Expression Analysis Systemic Explorer (EASE) scores of ≤0.05 were screened for functional annotation clustering.

#### Network Construction and Analysis

The compound-target network was generated by linking bioactive constituents and putative targets. The target-pathway network was established with the predicted targets and signaling pathways that were postulated to be involved in HCC. The compound-pathway network was constructed with all the compounds and the signaling pathways. In our network, the nodes represent the candidate compounds, potential targets, or signaling pathways, while the edges represent the compound-target or target-pathway interactions. The Cytoscape software was employed to construct the networks.

### Experimental Analyses

#### Chemicals and Reagents

HCSCs (Batch No. 7D05) was provided by Shanxi Eastantai Pharmaceutical Co., Ltd. Dulbecco’s Modified Eagle Medium (DMEM), fetal bovine serum (FBS), penicillin-streptomycin, and phosphate buffered saline (PBS) were purchased from Gibco. Thiazolyl Blue Tetrazolium Blue (MTT), paraformaldehyde (PFA), crystal violet solution, and propidium iodide (PI) were purchased from Sigma. Transwell chamber system, matrigel matrix, and FITC Annexin V/PI Apoptosis Detection kit were purchased from BD Bioscience.

#### Cell Lines and Cell Culture

The human HCC cell line, PLC/PRF/5, was purchased from ATCC (USA), while another human HCC cell line, MHCC97L, was a kind gift from Dr. Man Kwan, Department of Surgery, The University of Hong Kong. The cells were maintained in DMEM (Gibco, USA) with 10% FBS (Gibco, USA) and 1% penicillin-streptomycin (Gibco, USA). The cells were cultured and maintained at 37°C, and equilibrated with 95% air and 5% CO_2_. In addition, HCSCs (Batch No. 7D05) was provided by Shanxi Eastantai Pharmaceutical Co., Ltd, and stored at ambient temperature. The ingredient of HCSCs is toad skin. It was dissolved in PBS (Gibco, USA) in the experiments.

#### MTT Assay

The cell viability was measured with MTT assay. In brief, the cells were seeded onto 96-well plates, and treated with PBS or different dosages of HCSCs (0, 0.05, 0.1, 0.2, 0.4, 0.8, and 1.2 mg/ml) on the next day. After incubation for 48 h, 10 µl of MTT (Thiazolyl Blue Tetrazolium Blue; 5 mg/ml; Sigma, USA) was added to each well, followed by 4 h incubation at 37°C. The MTT was then discarded, and 100 µl of DMSO was added to each well. The absorbance of formazan formed was measured at 595 nm using a Multiskan MS microplate reader (Labsystems, Finland).

#### Clonogenic Assay

The cells were seeded onto six-well plates (10^4^ cells/well), and treated with indicated concentration of HCSCs for 12 d. At the end of the treatment, the medium was removed, and the cells were fixed using 4% paraformaldehyde (PFA; Sigma, USA) for 2 h, and stained with 0.1% crystal violet solution (Sigma-Aldrich, USA) for 30 min. Images were captured using an optical microscope, and clonogenic spheres were measured by manual counting in three random fields.

#### 3-D Transwell

The transwell invasion assay was conducted using a transwell chamber system (BD Biosciences, USA) with 8 µm pore. We firstly performed the proliferation assay with HCSCs treatment for 48 h, and then the survived population was collected and used for this assay. Briefly, the upper chamber was coated with matrigel matrix (BD, USA), and filled with 100 µl serum-free medium containing 5 × 10^4^ cells, while 500 µl DMEM containing 10% FBS and the indicated concentrations of HCSCs was added into the lower chamber. After incubation for 48 h, the non-invading cells on the upper surface of the chamber were removed using cotton swabs, and the cells, that have invaded across the matrigel matrix to the lower chamber, were fixed with 4% PFA and stained with 0.1% crystal violet solution. An inverted microscope at 200× magnification was used to image the stained cells in three random fields, and the cell number in each field was manually counted. The relative cell invasion rate was calculated by the number of invaded cells normalized to the total number of cells in the upper chamber.

#### Wound Healing Assay

The cells were cultured in 24-well plates until 100% confluence. A narrow section of the cells was removed using a sterile micropipette tip to create a wound of around 0.5 mm in width. The medium was then discarded, and the monolayer was gently rinsed twice using warm PBS. Next, the medium containing vehicle or indicated dosages of HCSCs was added to each well. The cell migration data were obtained with an inverted microscope (Olympus, Japan) at 0, 24, and 48 h incubation. The wound width of the cell-free area was also assessed by Image-J software (NIH, Bethesda, MD, USA).

#### Flow Cytometry

The cells were seeded onto six-well tissue culture plates and exposed to specified dosages of HCSCs for 48 h. For cell cycle analysis, the cells were harvested and fixed in 70% ethanol at 4°C overnight, followed by staining with propidium iodide (PI; Sigma-Aldrich, USA) in the dark for 15 min, and acquired with Canto II flow cytometer (BD Biosciences, USA). For the measurement of apoptosis, the cells were stained using the FITC Annexin V/PI Apoptosis Detection kit (BD Biosciences, USA) as per the manufacturer’s instructions. The percentage of apoptotic cells was calculated as the sum of percentages at Q2 and Q3. All the data were analyzed using the FlowJo software (BD, USA).

## Results

### Identification of Bioactive Components From HCSCs

**Figure 2:**
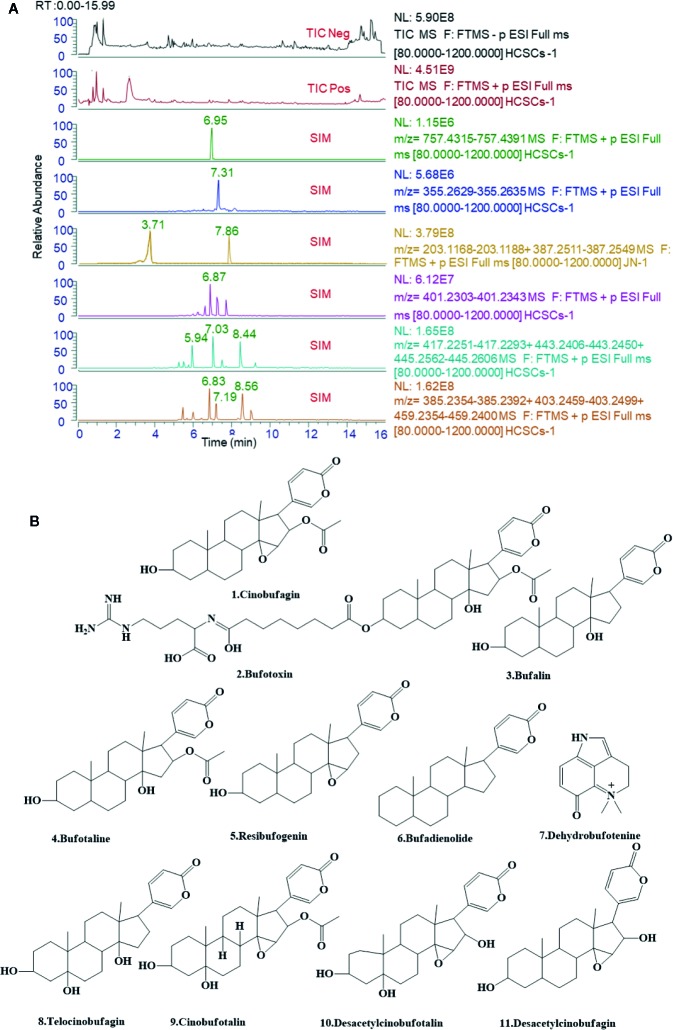
Ultra-performance liquid chromatography-tandem mass spectrometry (UPLC-MS/MS) analyses of Huachansu capsules (HCSCs) and their active compounds. **(A)** Total-ion chromatograms (TIC) and select-ion chromatograms (SIC) of HCSCs samples [Stationary phase: ACQUITY UPLC HSS T3 (2.1 mm×100 mm, 1.8 µm); mobile phase: 0.1% aqueous solution of formic acid **(A)** and acetonitrile **(B)** in gradient. The following gradient elution program was used: 0–12 min, 5%–98% B; 12–12.01 min, 98%–5% B; 12.01–15 min, 5% B; flow rate: 0.3 μl/min. (Peak No: Rt, 8.44 min, Cinobufagin; 6.95 min, Bufotoxin; 7.86 min, Bufalin; 7.03 min, Bufotalin; 8.56 min, Resibufogenin; 7.31 min, Bufadienolide; 3.71 min, Dehydrobufotenine; 6.83 min, Telocinobufagin; 7.19 min, Cinobufotalin; 5.94 min, Desacetylcinobufotalin; 6.87 min, and Desacetylcinobufagin. **(B)** The molecular formulas of the 11 bioactive components identified from Huachansu capsules (HCSCs).

The major components of HCSCs were separated and determined using UPLC-Q/ExactiveHybrid Quadrupole-Orbitrap system with the Compound Discoverer 2.1 software package (Thermo Finnigan, San Jose, CA, USA). As shown in **Figure 2**, phytochemical profile of HCSCs was detected, and 11 bioactive compounds were identified from HCSCs with chromatographic peaks. Then, mzCloud (ddMS2) and ChemSpider (exact mass or formula) were used to identify these compounds and search for their analogues. On the basis of their spectral data and chemical properties, 10 of the compounds were identified as nonsteroidal, including cinobufagin, bufotoxin, bufalin, bufotalin, resibufogenin, bufadienolide, telocinobufagin, cinobufotalin, desacetylcinobufotalin, and desacetylcinobufagin; the other compound was alkaloid, namely dehydrobufotenine. The details were shown in **Table 1** and **Figure 2**.

**Table 1:** Chemical formulae of the 11 bioactive components identified from Huachansu capsules (HCSCs).

No	Compound	Chemical formula	[M+H]^+^ Measured mass	[M+H]^+^ True mass	Accuracy (ppm)	RT (min)
1	Cinobufagin	C_26_H_34_O_6_	443.2428	443.2413	3.38	8.44
2	Bufotoxin	C_40_H_60_N_4_O_10_	757.4382	757.4353	3.83	6.95
3	Bufalin	C_24_H_34_O_4_	387.2517	387.2530	−3.36	7.86
4	Bufotalin	C_26_H_36_O_6_	445.2585	445.2570	3.37	7.03
5	Resibufogenin	C_24_H_32_O_4_	385.236	385.2373	−3.37	8.56
6	Bufadienolide	C_24_H_34_O_2_	355.2637	355.2631	1.69	7.31
7	Dehydrobufotenine	C_12_H_14_N_2_O	203.1175	203.1178	−1.48	3.71
8	Telocinobufagin	C_24_H_34_O_5_	403.2464	403.2479	−3.72	6.83
9	Cinobufotalin	_C26_H_34_O_7_	459.2362	459.2377	−3.27	7.19
10	Desacetylcinobufotalin	C_24_H_32_O_6_	417.2254	417.2272	−4.31	5.94
11	desacetylcinobufagin	C_24_H_32_O_5_	401.231	401.2323	−3.24	6.87

RT, retention time.

### HCC-Related Target Identification of HCSCs

Based on the 11 identified compounds, a total of 402 targets were collected from the SwissTargetPrediction, SEA, TCMSP, and STITCH databases, and there are 268 repetitive genes in 402 targets. The remaining 134 targets shared the common compounds. The detailed information was presented in **Supplementary Table 1**. Moreover, we collected HCC-associated human genes from five databases including OncoDB.HCC, Liverome, TTD, KEGG, CTD, and GeneCards, which presented specifically in **Supplementary Table 2**. According to the results presented in **Supplementary Tables 1** and **2**, the overlapping genes for compound targets and HCC-associated human genes was obtained by using Venny 2.1.0 (Liang et al., 2019). As a result, 82 HCC-related genes were identified for the 11 components of HCSCs (**Table 2**).

**Table 2:** Hepatocellular carcinoma-related targets of Huachansu capsules (HCSCs).

Number	Protein name	Gene name
01	Steroid 5 Alpha-Reductase 1	SRD5A1
02	Aldo-Keto Reductase Family 1 Member B10	AKR1B10
03	Nuclear Receptor Subfamily 3 Group C Member 1	NR3C1
04	Solute Carrier Family 10 Member 1	SLC10A1
05	Vitamin D Receptor	VDR
06	Tyrosine Aminotransferase	TAT
07	Hydroxysteroid 11-Beta Dehydrogenase 2	HSD11B2
08	Androgen Receptor	AR
09	ATPase Na^+^/K^+^ Transporting Subunit Alpha 1	ATP1A1
10	Solute Carrier Family 10 Member 2	SLC10A2
11	UDP Glucuronosyltransferase Family 2 Member B7	UGT2B7
12	Cytochrome P450 Family 27 Subfamily B Member 1	CYP27B1
13	Cytochrome P450 Family 3 Subfamily A Member 4	CYP3A4
14	Microtubule-associated protein tau	MAPT
15	Opioid Receptor Mu 1	OPRM1
16	Opioid Receptor Delta 1	OPRD1
17	Opioid Receptor Kappa 1	OPRK1
18	Muscleblind Like Splicing Regulator 2	MBNL2
19	Muscleblind Like Splicing Regulator 3	MBNL3
20	Butyrylcholinesterase	BCHE
21	Acetylcholinesterase (Cartwright Blood Group)	ACHE
22	ATP Binding Cassette Subfamily B Member 11	ABCB11
23	Serpin Family A Member 6	SERPINA6
24	Glucose-6-Phosphate Dehydrogenase	G6PD
25	Gamma-Aminobutyric Acid Type B Receptor Subunit 1	GABBR1
26	G Protein-Coupled Bile Acid Receptor 1	GPBAR1
27	Nuclear Receptor Subfamily 1 Group H Member 4	NR1H4
28	Nuclear Receptor Subfamily 1 Group I Member 3	NR1I3
29	Sex Hormone Binding Globulin	SHBG
30	Bradykinin Receptor B2	BDKRB2
31	ATP Binding Cassette Subfamily C Member 4	ABCC4
32	ST6 Beta-Galactoside Alpha-2,6-Sialyltransferase 1	ST6GAL1
33	Matrix Metallopeptidase 3	MMP3
34	Matrix Metallopeptidase 1	MMP1
35	Matrix Metallopeptidase 10	MMP10
36	Matrix Metallopeptidase 8	MMP8
37	Matrix Metallopeptidase 12	MMP12
38	Matrix Metallopeptidase 13	MMP13
39	Growth Factor, Augmenter Of Liver Regeneration	GFER
40	Angiotensin I Converting Enzyme	ACE
41	Angiotensin I Converting Enzyme 2	ACE2
42	ADAM Metallopeptidase Domain 17	ADAM17
43	Carboxyl Ester Lipase	CEL
44	Cytochrome P450 Family 17 Subfamily A Member 1	CYP17A1
45	Matrix Metallopeptidase 2	MMP2
46	Eukaryotic Translation Initiation Factor 2 Alpha Kinase 3	EIF2AK3
47	Caspase 3	CASP3
48	MYB Proto-Oncogene, Transcription Factor	MYB
49	Poly(ADP-Ribose) Polymerase 1	PARP1
50	Interferon Regulatory Factor 3	IRF3
51	Cytochrome P450 Family 3 Subfamily A Member 5	CYP3A5
52	TNF Receptor Superfamily Member 10a	TNFRSF10A
53	Glycogen Synthase Kinase 3 Beta	GSK3B
54	Cannabinoid Receptor 1	CNR1
55	Estrogen Receptor 1	ESR1
56	Estrogen Receptor 2	ESR2
57	Cannabinoid Receptor 2	CNR2
58	Carbonic Anhydrase 1	CA1
59	Carbonic Anhydrase 2	CA2
60	Nuclear Receptor Subfamily 3 Group C Member 2	NR3C2
61	Sonic Hedgehog	SHH
62	Histone Deacetylase 1	HDAC1
63	Histone Deacetylase 3	HDAC3
64	Histone Deacetylase 2	HDAC2
65	Fatty Acid Binding Protein 1	FABP1
66	Progesterone Receptor	PGR
67	Solute Carrier Family 22 Member 2	SLC22A2
68	Steroid 5 Alpha-Reductase 2	SRD5A2
69	Hydroxysteroid 17-Beta Dehydrogenase 12	HSD17B12
70	5-Hydroxytryptamine Receptor 2B	HTR2B
71	Epidermal Growth Factor Receptor	EGFR
72	Erb-B2 Receptor Tyrosine Kinase 2	ERBB2
73	Erb-B2 Receptor Tyrosine Kinase 4	ERBB4
74	Erb-B2 Receptor Tyrosine Kinase 3	ERBB3
75	Kruppel Like Factor 5	KLF5
76	Carbonic Anhydrase 3	CA3
77	Secreted Frizzled Related Protein 1	SFRP1
78	Coagulation Factor II Thrombin Receptor	F2R
79	Monoglyceride Lipase	MGLL
80	Galactosidase Beta 1	GLB1
81	Histone Deacetylase 6	HDAC6
82	Solute Carrier Family 5 Member 1	SLC5A1

### Compound-Target Network Analysis

**Figure 3:**
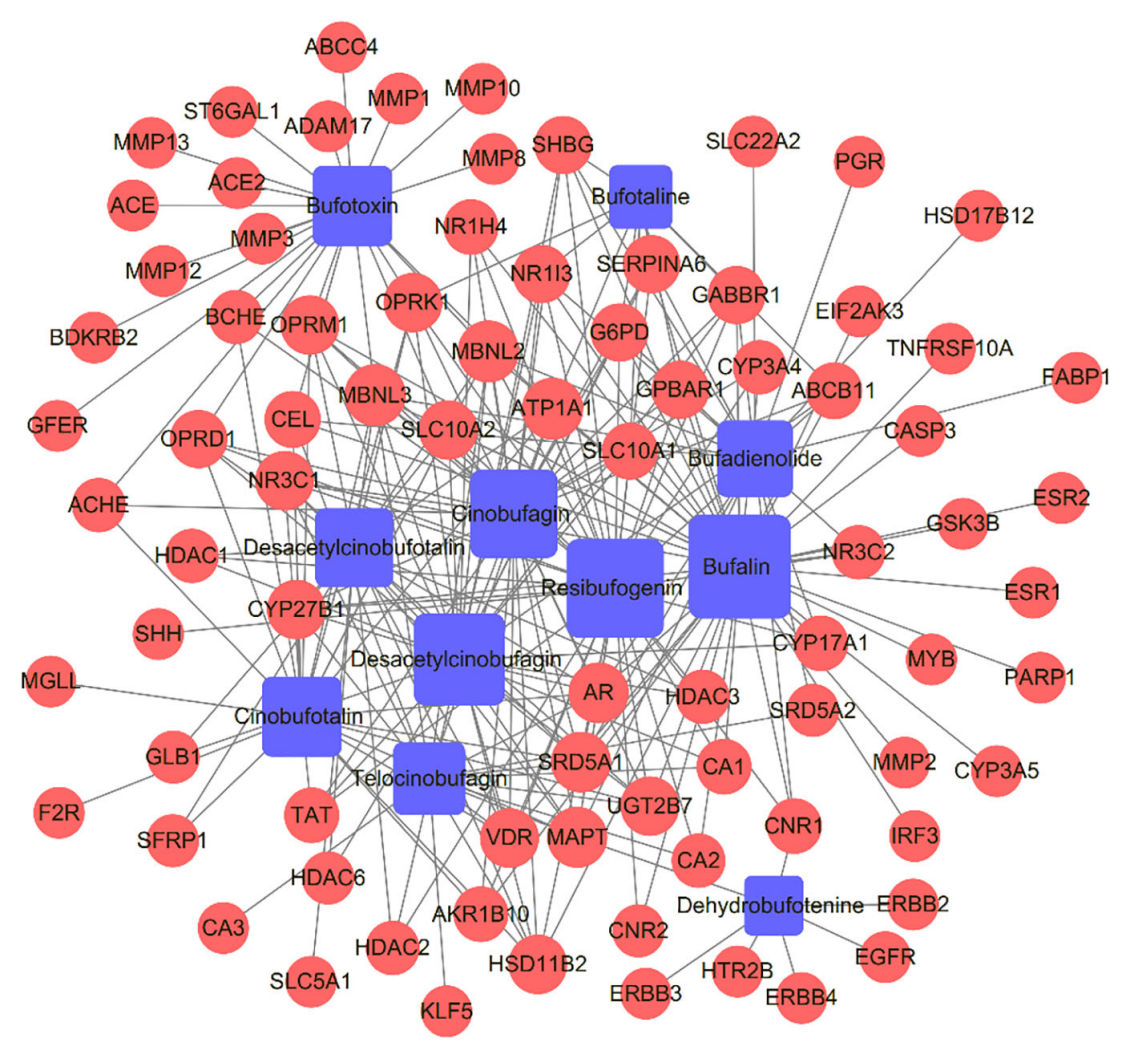
Compound-target network of Huachansu capsules (HCSCs) and hepatocellular carcinoma. The blue nodes represent candidate active compounds, and the red nodes represent potential protein targets. The edges represent the interactions between them, and the node sizes are proportional to the node degrees.

Chinese herbal formulas present a range of pharmacological activities through a range of targets, so we investigated the potential mechanisms of HCSCs against HCC. Based on the compounds and predicted targets, we constructed a network of components and targets using Cytoscape. The centralization and heterogeneity of the network were 0.393 and 1.400, respectively. As presented in **Figure 3**, the network contained a total of 93 nodes, 11 compound nodes and 82 target nodes, which formed 261 compound-target associations. The network indicated the potential relationships between the compounds and the targets, thereby revealing the potential pharmacological mechanisms of HCSCs for the treatment of HCC. The nodes with the highest degree of connections to other compounds or targets represented hubs within the entire network, and hence were potential drugs or targets. For example, the compound with the highest degree of connections was bufalin (degree=41). Resibufogenin, desacetylcinobufagin, and cinobufagin also have higher degree of connections of 37, 32, and 29, respectively. These findings indicated that a single compound affected multiple targets, and these targets were potentially related to action of HCSCs. In terms of target analysis, SRD5A1, AR, and MAPT individually linked to 56 compounds; ATP1A1, MBNL2, and MBNL3 were individually connected to nine compounds; NR3C1, VDR, HSD11B2, SLC10A2, UGT2B7, CYP27B1, OPRM1, and OPRK1 individually linked to seven compounds; OPRD1, SERPINA6, G6PD, GABBR1, GPBAR1, NR1I3, and SHBG connected to six compounds, respectively. These findings indicated that multiple compounds could target a single gene in an interactional manner, supporting that HCSCs exhibited inhibitory function through multi-components and multi-target treatment.

### PPI Network of HCSC-Related HCC Targets

**Figure 4:**
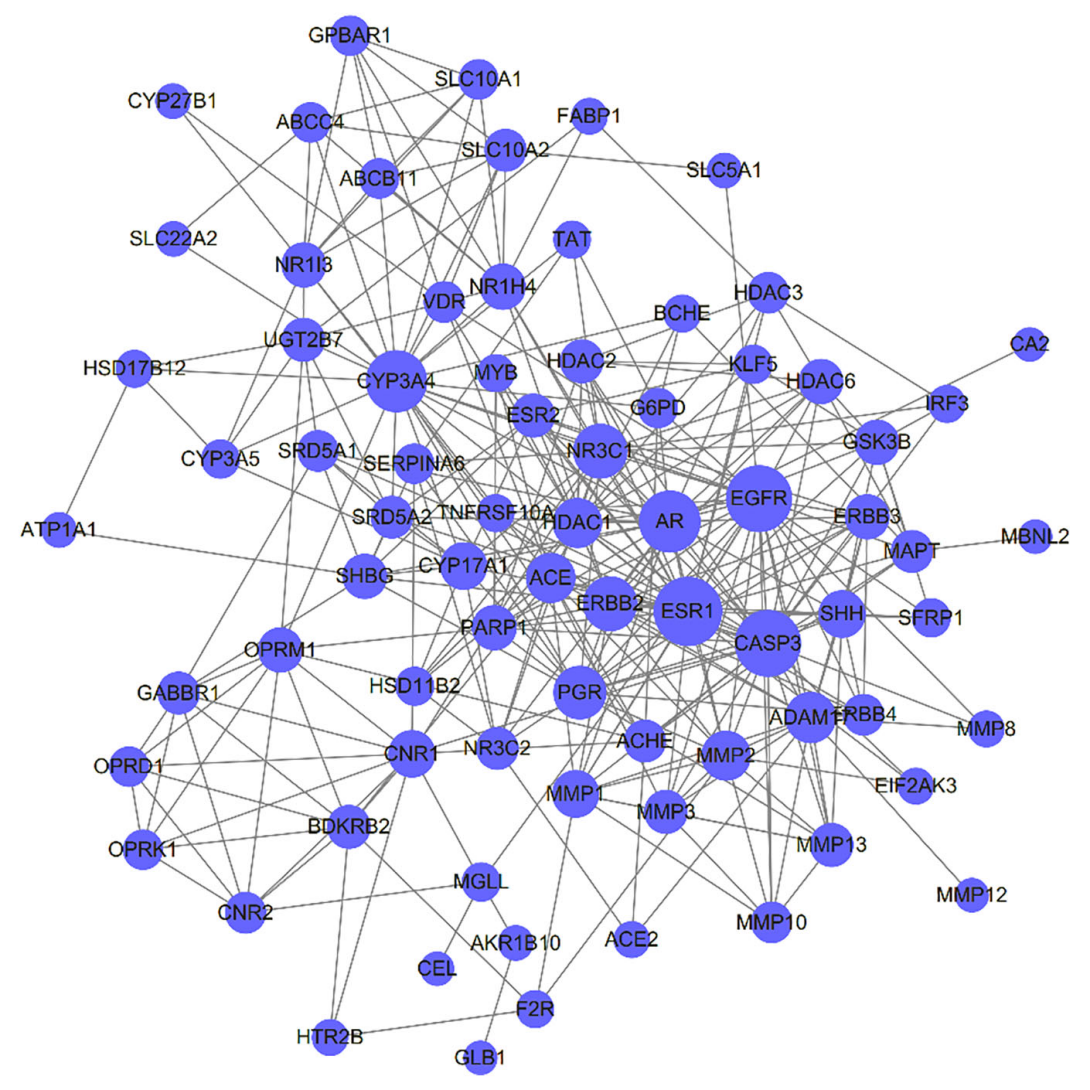
Protein-Protein Interaction (PPI) network of Huachansu capsules (HCSCs)-related targets in hepatocellular carcinoma. The size of the nodes is proportional to the degree of the nodes.

The PPI network was constructed based on the PPIs for the candidate protein targets of HCSCs. **Figure 4** showed that the PPI network consisted of 77 nodes and 324 edges. The centralization and heterogeneity of the network were 0.292 and 0.751, respectively. In the PPI network, the nodes with higher degree might play important roles in the pharmacological processes. It demonstrated that 10 key nodes, including ESR1, CASP3, EGFR, AR, CYP3A4, ERBB2, NR3C1, PGR, ADAM17, and MMP2 were likely to be the key targets of HCSCs to inhibit HCC (**Supplementary Table 3**).

### GO and KEGG Pathway Enrichment Analyses

**Figure 5:**
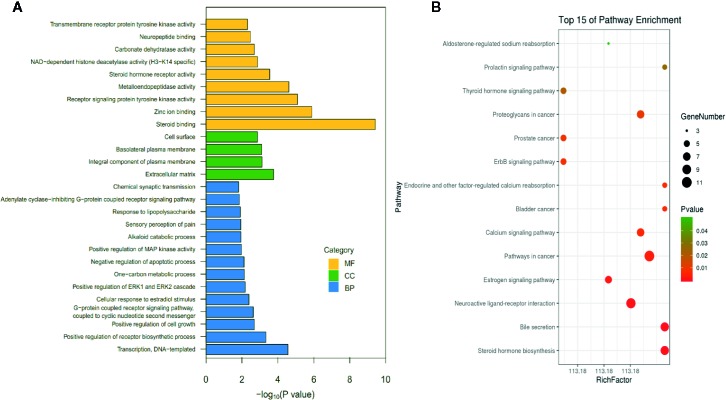
List of Gene Ontology (GO) and Kyoto Encyclopedia of Genes and Genomes (KEGG) pathway enrichment results in relation to the potential targets of Huachansu capsules (HCSCs). **(A)** The first 27 GO terms were identified based on *p* < 0.01. **(B)** The top 15 pathways were identified based on *p* < 0.05.

To verify the biological characteristics of involved target genes of HCSCs against HCC, the GO enrichment analysis of putative targets was performed using DAVID Bioinformatics Resources, a type of functional annotation tool. Among the 77 genes, 68 GO terms met the requirements with the count of ≥2 and EASE scores of ≤0.05, in which 31 items were BP-related, 10 items were CC-related, and 27 were MF-related. The GO information was presented in detail in **Supplementary Table 4**. **Figure 5A** showed the top 27 enriched terms in BP, CC, and MF categories, suggesting that HCSCs may regulate cancer cell proliferation through protein, enzyme, and transcription factor binding in the extracellular space, plasma membrane, or cytosol, to exhibit inhibitory effects in HCC.

**Figure 6:**
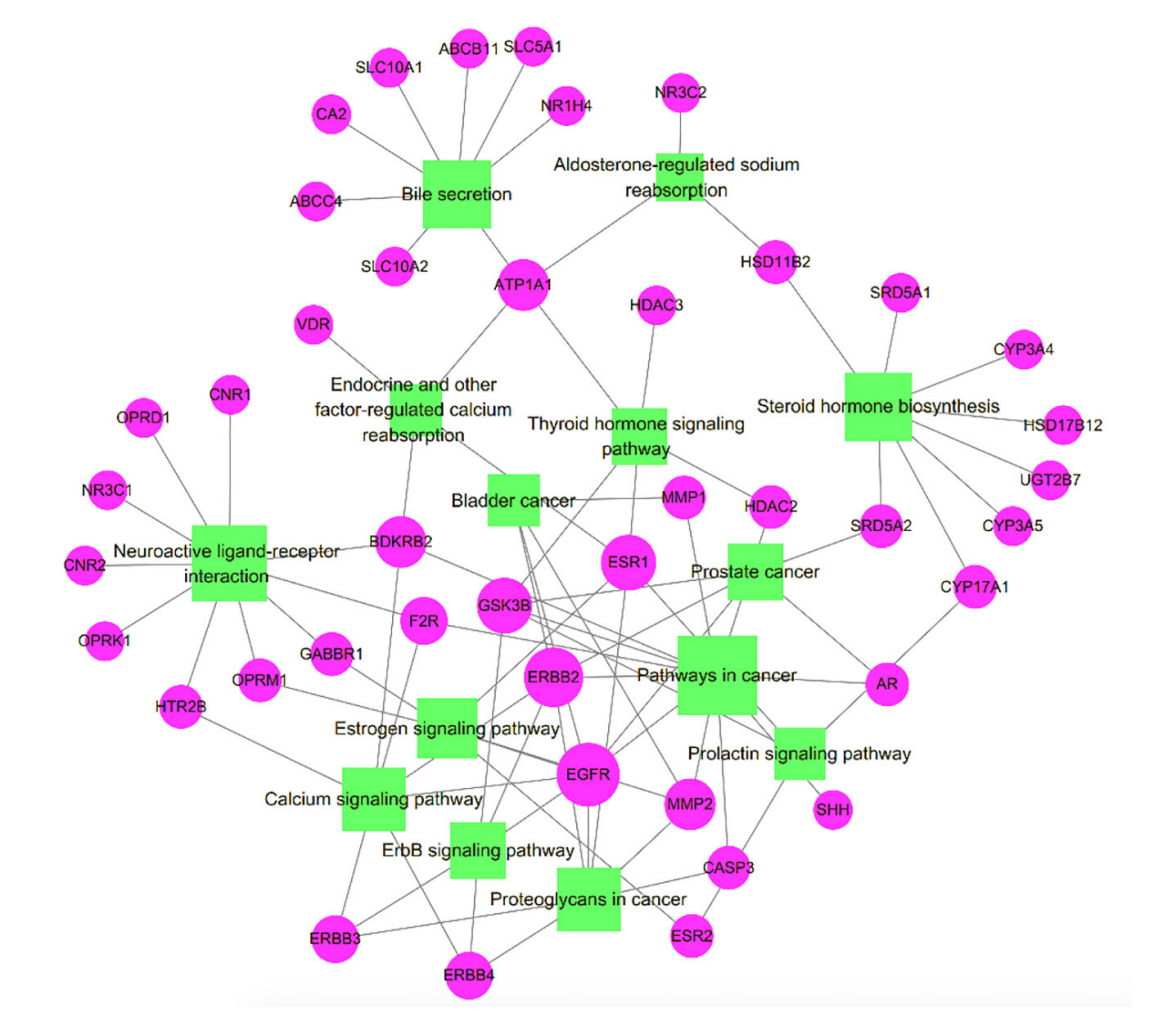
Target-pathway network of Huachansu capsules (HCSCs). The green nodes represent the pathways, the pink nodes represent the targets, and the edges represent the interactions between them. The size of the node is proportional to the degree of the node.

To further identify the potential pathways involved in the inhibitory effects of HCSCs against HCC, the KEGG pathway enrichment analysis of the 77 genes was performed. As shown in **Figure 5B**, a total of 14 enriched pathways of HCSCs against HCC were identified (p < 0.05). The KEGG pathway information was presented in detail in **Supplementary Table 5**. The enriched genes were linked to a variety of pathways, including pathways in cancer, bladder cancer, prostate cancer, proteoglycans in cancer, as well as metabolic, immune, and apoptotic pathways. To further clarify the modes of action, a target-pathway network was constructed based on all these target proteins and the corresponding signaling pathways. The centralization and heterogeneity of the network were 0.149 and 0.804, respectively. As shown in **Figure 6**, this network was composed of 87 edges and 56 nodes, including 14 for pathways and 42 for proteins. Among these protein targets, ERBB2, EGFR, ESR1, GSK3B, MMP2, CASP3, ATP1A1, BDKRB2, and AR were identified as relatively high-involved molecules, which suggested that these proteins may play essential roles in HCC progression.

**Figure 7:**
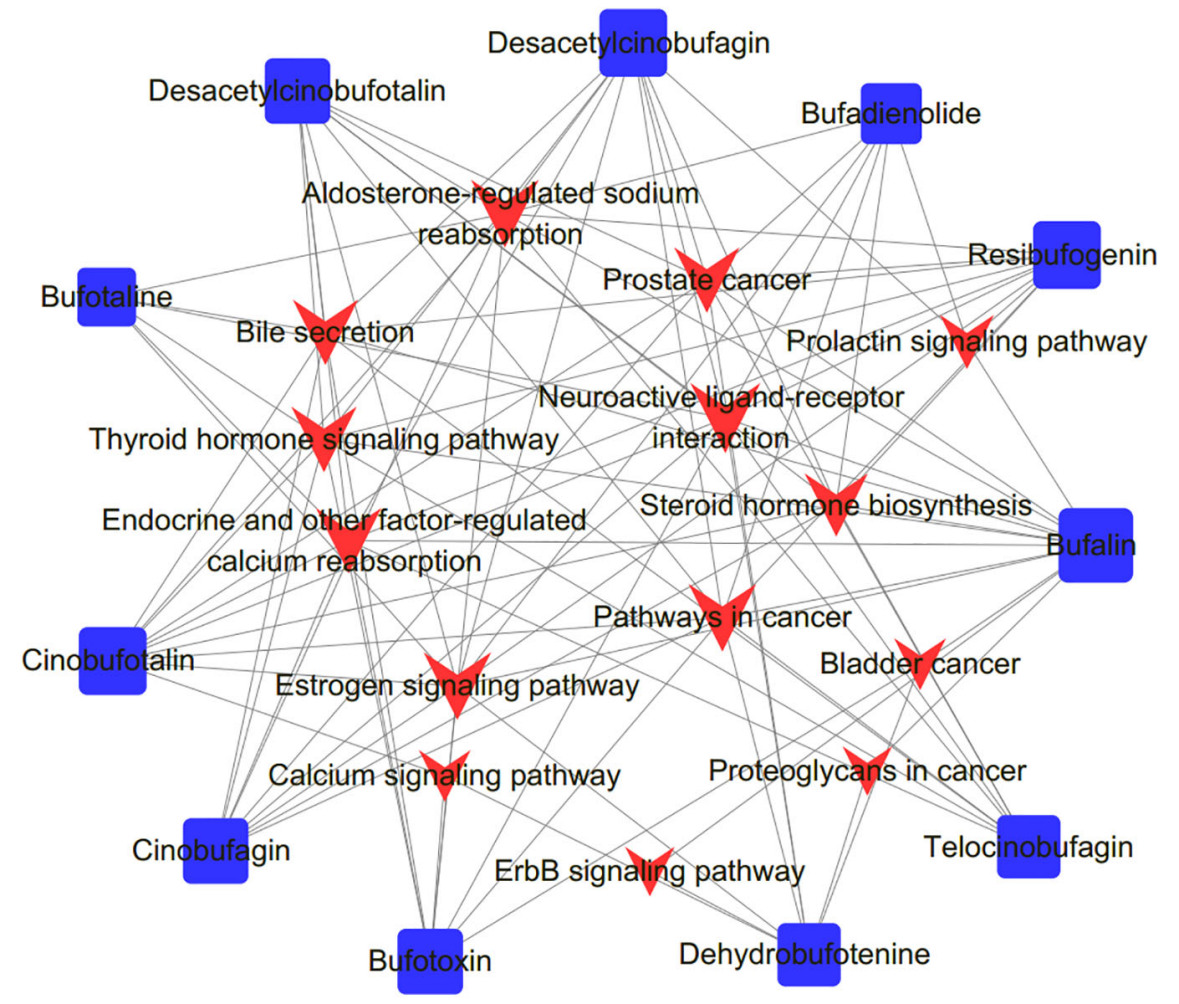
Compound-pathway network of Huachansu capsules (HCSCs). The red nodes represent significant pathways, the blue nodes represent candidate active compounds, and the edges represent the interactions between them. The size of the node is proportional to the degree of the node.

To further clarify the pathways that regulated by the compounds, a compound-pathway network was constructed based on all the compounds and signaling pathways. The centralization and heterogeneity of the network were 0.230 and 0.364, respectively. As shown in **Figure 7**, the network was composed of 25 nodes (14 pathways and 11 compounds) and 99 edges. Taken together, we suggested that HCSCs exerted anti-tumor effects against HCC through multiple mechanisms.

### HCSCs Inhibited HCC Proliferation

**Figure 8:**
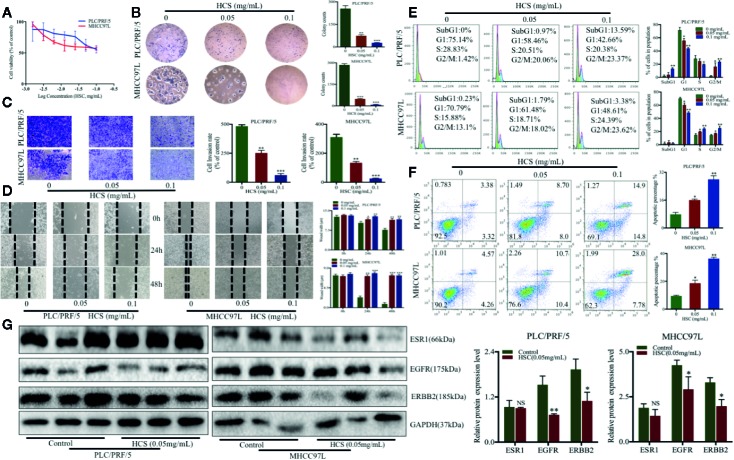
Huachansu capsules (HCSCs) exhibited anti-cancer effects in PLC/PRF/5 and MHCC97L cells. **(A)** The cell viability was assessed by MTT assay after HCSCs administration for 48 h. The cells were incubated with 0–1.2 mg/ml HCSCs. n=3. **(B)** Colony formation in the presence of HCSCs. n=3. **(C)** Transwell invasion abilities of HCC cells in the presence of HCSCs. n=3. **(D)** Wound healing abilities of HCC cells in the presence of HCSCs. n=3. **(E)** Cell cycle analysis in HCSCs-treated HCC cells. n=3. **(F)** Apoptosis analysis of HCSCs-treated HCC. n=3. **(G)** Western blot analysis of ESR1, EGFR, and ERBB2 in HCC cells after 48 h treatment with HCSCs of 0.05 mg/ml. **(B–G)** The cells were treated with 0.05 and 0.1 mg/ml HCSCs for 48 h. ^*^ *p* < 0.05, ^**^ *p* < 0.01, ^***^ *p* < 0.001 vs. 0 mg/ml.

To validate the effects of HCSCs against HCC as predicted from network pharmacology analyses, a series of cell biological function assays were performed. MTT assay was conducted to identify the effects of HCSCs on cell viability in HCC, PLC/PRF/5, and MHCC97L cells. After exposure to 0–1.2 mg/ml of HCSCs for 48 h, the HCC cells were observed a dose-dependent decrease in cell viability (**Figure 8A**). The IC_50_ (the half maximal inhbitory concentration) values of HCSCs were 0.1132 and 0.1807 mg/ml for PLC/PRF/5 and MHCC97L cells, respectively.

Next, we examined the anti-cancer effects of HCSCs in PLC/PRF/5 and MHCC97L cells. The cells were exposed to HCSCs (0.05 and 0.1 mg/ml) for 48 h. The colony formation assay was performed to examine the colony formation abilities of the HCC cells in the presence of HCSCs. The colony counts of PLC/PRF/5 and MHCC97L cells were significantly reduced by HCSCs (**Figure 8B**). Moreover, the transwell invasion assay was also carried out to measure the invasive ability of the HCC cells in the presence of HCSCs. After incubation with HCSCs, the invasive abilities of the HCC cells were significantly reduced (**Figure 8C**). Likewise, we observed that HCSCs significantly increased the wound width in HCC cells (**Figure 8D**), indicating its wound healing abilities. Interestingly, the cell cycle arrest at the G2/M stage was observed in HCC cells (**Figure 8E**). Furthermore, the apoptotic profile showed that HCSCs significantly induced apoptosis in HCC cells (**Figure 8F**). To further validate the feasibility of pharmacology network analysis, we selected the top proteins with high degree from PPI network results and found that the proteins expression of EGFR, ERBB2 were significantly decreased after HCSCs treatment, but ESR1, a tumor suppression gene, had no obvious changes after HCSCs treatment (**Figure 8G**). We reasoned that HCSCs inhibited HCC growth in part through inhibiting ERBB, EGFR signaling pathways. Taken together, this indicated that HCSCs have strong anti-cancer effects in HCC.

## Discussion

HCC is one of the main types of liver cancer, which has high incidence and mortality around the world. According to GLOBOCAN 2018 China, Southeastern Asia, Eastern Africa, and sub-Saharan western have the highest incidence of HCC. Up to now, there is still lack of effective medicine to inhibit the progression of HCC, so new effective agents are needed to be developed. According to the principles of Chinese medicine, liver cancer is being considered as the cumulative toxicity of internal organs that combines several patterns of syndrome. Chinese herbal formulas are composed of multi-components for treating complex symptoms in liver cancer. Being made up of various components, Chinese herbal formulas play a variety of functions through synergistic or contradictory actions between multiple compositions (Wang et al., 2014). The multiple compositions may be responsible for multi-targets and -pathways underlying the effects of Chinese herbal formulas in liver cancer. Considering that the underlying mechanisms of Chinese herbal formulas in diseases are difficult to be clarified (Liu and Du, 2010), network pharmacology analysis may shed the light to the study of the mechanisms of Chinese herbal formulas. This analysis can predict the target profiles and pharmacological mechanisms of the active constituents in Chinese herbal formulas, thereby suggesting some interactions between drugs and organisms at a systematic level (Li and Zhang, 2013). In this study, we identified the chemical compounds with biological activity from HCSCs and the possible targets and pathways in relation to HCC, and also investigated the anti-cancer effects of HCSCs in HCC, PLC/PRF/5, and MHCC97L cells.

Huachansu, one type of Chinese medicine derived from dried toad venom, has been shown to display efficacy for the treatment of several cancers such as gastric and bladder cancer (Xie et al., 2013; Yang et al., 2015; Ni et al., 2019). A study demonstrated that with the combination of cinobufacin, one of the bioactive compounds from Huachansu, the transcatheter arterial chemoembolization could significantly improve the life quality and increase the 2-year survival rate in advanced HCC patients (Wu et al., 2014). Chinese herbal formulas are widely used in China to treat diseases. HCSCs, a formula of Huachansu, has been produced and consumed in China for many years. In this study, 11 chemical components of HCSCs were identified using UPLC-MS/MS. Among these 11 bioactive components, several compounds have been reported to possess pharmacological actions against cancers including HCC. Previous work reported that bufotalin and telocinobufagin were promising anti-HCC agents (Zhang et al., 2012), while cinobufotalin was a potential anti-hepatoma component (Cheng et al., 2015). In addition, bufalin (Qiu et al., 2013), cinobufagin (Qi et al., 2011), resibufogenin (Xie et al., 2012), and bufadienolide (Wei et al., 2017) are the main anti-cancer components of Huachansu injection.

The network pharmacology analysis may predict the potential targets and pathways underlying the anti-cancer effects of HCSCs against HCC. We applied a series of databases to retrieve HCC-related targets for these 11 bioactive compounds of HCSCs. The PPI network analysis was used to identify key proteins related to the anti-HCC effects of HCSCs, which were implied by the crucial nodes in the PPI network. The top 10 key targets, namely ESR1, CASP3, EGFR, AR, CYP3A4, ERBB2, NR3C1, PGR, ADAM17, and MMP2, were demonstrated as hub-bottleneck proteins according to the topological parameters. The interactive structure indicated that HCSCs could interact directly or indirectly with these molecular targets to exert anti-cancer effects. We also observed that ESR1 connected 30 nodes in the PPI network. ESR1 encodes the estrogen receptor, which is important for hormone binding, DNA binding, and transcription activation. The silence of ESR1 significantly increased the size of tumor in HCC tissues, implying that ESR1 is a tumor suppressor in human HCC (Hishida et al., 2013). Moreover, EGFR, a transmembrane receptor tyrosine kinase, connected 28 nodes in the PPI network. The activation of EGFR could lead to the activation of diverse signaling pathways that regulate cell proliferation, differentiation, and survival (Komposch and Sibilia, 2015). CASP3, a type of cysteinyl aspartate-specific proteases, functions as an important effector in apoptotic process (Hong et al., 2017), while CYP3A4 encodes a member of the cytochrome P450 superfamily of enzymes and is used as a biomarker for HCC prognosis (Ashida et al., 2017). Therefore, we concluded that these proteins are important for tumor-suppressing activity of HCSCs in HCC.

Sixty-eight GO terms and 14 KEGG pathways were identified using enrichment analysis. Our results indicated that HCSCs may exert anti-tumor effects through the dysregulation of HCC cell proliferation, which characterize the potential mode of action of HCC progression. Based on a series of network pharmacology analyses, HCSCs may exhibit inhibitory actions in HCC *via* directly modulating cancer, metabolism and immune-related pathways. For example, the ERBB signaling pathway is involved in multiple human cancers, and signaling through the ERBB/HER receptors already serves as a target for several cancer drugs (Citri and Yarden, 2006). This is in consistence with our compound-pathway network analysis showing that the ERBB signaling pathway was a potential underlying mechanism for bufalin and dehydrobufotenine. Moreover, among these 14 pathways, bufalin was involved in 13 pathways of them, resibufogenin, desacetylcinobufagin, and cinobufotalin was involved in 10 pathways, and other compounds involved in at least six pathways. It also showed that the cancer pathways were linked with 10 compounds, and the remaining pathways interacted with at least two compounds. Taken together, we suggested that HCSCs regulate multiple targets and pathways in HCC cells.

To further verify the anti-HCC ability of HCSCs, a series of biological function assays were performed in HCC cell lines, PLC/PRF/5 and MHCC97L. We showed that HCSCs induced cell death and cell cycle arrest at G2/M phase in HCC cells. Moreover, we found that HCSCs inhibited colony formation, cell invasion and migration, and apoptosis in HCC cells. This is in consistent with a previous study of gastric cancer, which reported that HCSCs has significant anti-proliferative and apoptotic effects in gastric cancer cells (Ni et al., 2019).

Taken together, we identified 11 bioactive components in HCSCs and the potential targets and pathways underlying the effects of HCSCs in HCC using the network pharmacology method. The results identified some pathways and biological processes that could be related to anti-cancer effects of HCSCs against HCC. To further validate the feasibility of pharmacology network analysis, we selected the top proteins with high degree from PPI network results and found that the proteins expression of EGFR, ERBB2 were significantly decreased after HCSCs treatment, but ESR1, a tumor suppression gene, had no obvious changes after HCSCs treatment. We reasoned that HCSCs inhibited HCC growth in part through inhibiting ERBB, EGFR signaling pathways. Therefore, this study provides an alternative method for the comprehensive understanding of the anti-HCC mechanisms of HCSCs.

## Conclusion

In conclusion, the current study identified, for the first time, the bioactive components in HCSCs, as well as the multiple targets and pathways of HCSCs against HCC. The network pharmacological method developed and the experimental evaluations in this study pointed to a new window for laboratory research and clinical application of HCSCs for the treatment of HCC.

## Data Availability Statement

Publicly available datasets were analyzed in this study. This data can be found here: OncoDB.HCC (http://oncodb.hcc.ibms.sinica.edu.tw/index.htm), Liverome (http://liverome.kobic.re.kr/index.php), Therapeutic Target Database (TTD, http://bidd.nus.edu.sg/group/cjttd/TTD_HOME.asp), Comparative Toxicogenomics Database (CTD, http://ctdbase.org/), PharmGKB (https://www.pharmgkb.org/), DAVID Bioinformatics Resources 6.7 (http://david.abcc.ncifcrf.gov/), and Chinese medicine systems pharmacology (TCMSP) database (http://lsp.nwsuaf.edu.cn/tcmsp.php).

## Author Contributions

YF and JH designed and conceived the study. JH and FC conceived the study and drafted the manuscript. ZZ, HT, NW, YL, and XF retrieved and analyzed the data. ZZ and TY revised the manuscript. All authors have read and approved the final manuscript.

## Funding

This study was supported by the Shanghai scientific and technological innovation action plan in 2017(17401970900); the China Postdoctoral Science Foundation funded project (2017M622811); the Natural Science Foundation of Guangdong Province, China (No. 2018A030310226).

## Conflict of Interest

The authors declare that the research was conducted in the absence of any commercial or financial relationships that could be construed as a potential conflict of interest.
