# Supplementary figures and images for: Interpreting the Pharmacological Mechanisms of Huachansu Capsules on Hepatocellular Carcinoma Through Combining Network Pharmacology and Experimental Evaluation
Source: Front Pharmacol. 2020 Apr 3;11:414. doi: 10.3389/fphar.2020.00414 (PMC7145978; doi:10.3389/fphar.2020.00414)

PLC/PRF/5

MHCC97L

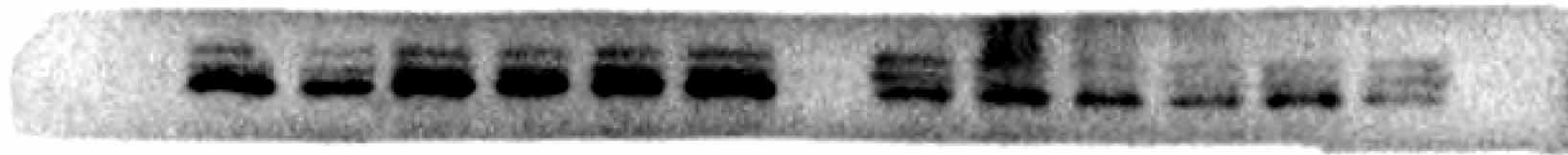

ESR1(66kDa)  
(Membrane 1)

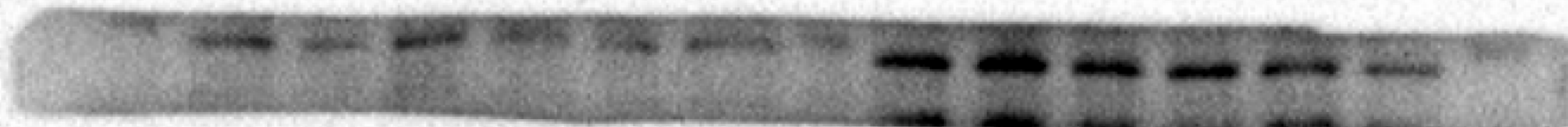

EGFR(175kDa)  
(Membrane 1)

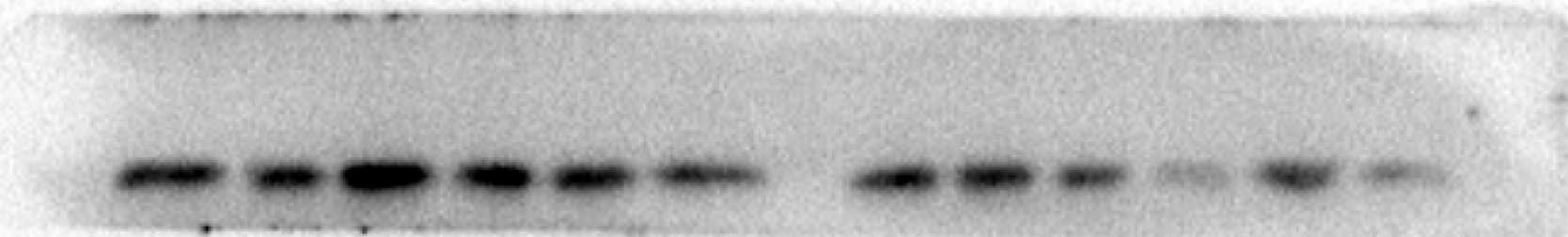

ERBB2(185kDa)  
(Membrane 2)

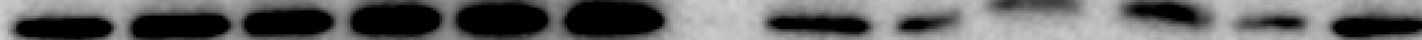

GAPDH(37kDa)  
(Membrane 1)

Supplement: Supplementary file 6 [file DataSheet_1.pdf]
